# The effects of essential oil mouthrinses with or without alcohol on plaque and gingivitis: a randomized controlled clinical study

**DOI:** 10.1186/s12903-017-0454-6

**Published:** 2018-01-10

**Authors:** Michael C. Lynch, Sheila Cavalca Cortelli, James A. McGuire, Jane Zhang, Danette Ricci-Nittel, Carolyn J. Mordas, Davi Romeiro Aquino, Jose Roberto Cortelli

**Affiliations:** 1 Johnson & Johnson Consumer Inc.,, 199 Grandview Road, Skillman, New Jersey 08558 USA; 2 Nucleus of Periodontal Research, Dental School, University of Taubate, Taubaté, São Paolo Brazil

**Keywords:** Essential oils, Mouthrinse, Alcohol-free, Plaque, Gingival bleeding, Gingivitis

## Abstract

**Background:**

The efficacy of several variants of essential oil mouthrinses has been studied extensively. This is the first study to compare the anti-plaque and anti-gingivitis efficacy of two marketed essential oil mouthrinses: one is an alcohol containing mouthrinse and the other one is an alcohol-free mouthrinse.

**Methods:**

This examiner-blind, parallel-group study randomized subjects to three groups: 1) Mechanical Oral Hygiene (MOH) only; 2) MOH plus Alcohol-Containing essential oil Mouthrinse (ACM); 3) MOH plus Alcohol-Free essential oil Mouthrinse (AFM). Primary endpoint was whole-mouth mean Modified Gingival Index (MGI) at six months. Secondary endpoints included whole-mouth mean MGI at one and three months, and whole-mouth mean Plaque Index (PI) and whole-mouth mean Bleeding Index (BI) at one, three and six months. Safety assessments were conducted at all time points.

**Results:**

A total of 370 subjects were enrolled; 348 subjects completed the study. After six months, subjects using essential oil mouthrinses with or without alcohol showed significant reduction (*p* < 0.001) in gingivitis (28.2% and 26.7%, respectively) and significant reduction (*p* < 0.001) in plaque (37.8% and 37.0%, respectively), compared to those performing MOH only. Significant reductions in MGI, PI, and BI (*p* < 0.001) were observed at one and three months and also at six months for mean BI. No statistically significant differences were observed for all measured indices between ACM and AFM groups at any time point. Both mouthrinses were well tolerated.

**Conclusions:**

No significant differences were observed in the efficacy of ACM and AFM to reduce plaque and gingivitis, when used in addition to MOH, over six months.

**Trial registration:**

The trial was registered on clinicaltrials.gov on November 30, 2016. The registration number is NCT02980497.

## Background

When used in conjunction with mechanical oral hygiene (MOH), the use of mouthrinses to help reduce plaque and gingivitis has been well documented and their efficacy is attributed to chemical agents, such as Cetylpyridinium Chloride (CPC), Chlorhexidine (CHX), and Essential Oils (EO) contained in these products [1–9]. Commonly used inactive ingredients include alcohol, water, buffering agents, flavouring agents and surfactants. In EO mouthrinses, alcohol serves as a preservative but also solubilizes the essential oils, thus maintaining their bioavailability [10, 11]. A 2015 meta-analysis of 29 clinical trials with durations of six months or longer further demonstrated the efficacy of essential oil mouthrinses [7].

The inclusion of alcohol in mouthrinse has historically limited its use in certain patient populations, such as children, alcoholics, people with strong taste preferences, those of certain religious beliefs, and patients with oral mucositis [12].

To address these limitations, an Alcohol-Free EO-mouthrinse (AFM) was developed. Its antimicrobial properties were demonstrated in vitro [13] and in vivo [14] initially. This was followed by demonstration of its efficacy in reducing plaque and gingivitis in clinical trials [15, 16]. However, no long-term studies (i.e., at least six months in duration) have directly compared alcohol containing EO mouthrinses (ACM) to its Alcohol-Free counterpart, AFM. The objective of the current study is to compare the long-term efficacy of twice-daily rinsing with either an AFM or an ACM, in conjunction with usual MOH, for the reduction of plaque and gingivitis.

## Methods

### Study design

This randomized, controlled, single-centre, examiner-blind, parallel-group study was conducted between 11 September 2013 and 16 March 2014 (recruitment period July 2013–September 2013) at the University of Taubate, São Paulo, Brazil. The study was conducted in accordance with the International Conference on Harmonisation (ICH) Harmonised Tripartite Guideline for Good Clinical Practice (ICH E6, 1996), in agreement with the Declaration of Helsinki (2000), applicable local regulations, and the American Dental Association (ADA) Seal of Acceptance Program Guidelines for Chemotherapeutic Products for Control of Gingivitis [17]. The study protocol was approved by the Institutional Ethics Committee on research involving humans (CAAE: 20,402,713.1.00005501). The trial was registered on clinicaltrials.gov. The registration number is NCT02980497. Written informed consent was obtained from all subjects.

### Subjects

Subjects from Taubate and neighbouring areas were selected from a database or recruited through advertising and were consecutively enrolled in the study. Males and females aged ≥18 years, in good general and oral health, with the exception of mild to moderate gingivitis, and with a minimum of 20 natural teeth having scorable facial and lingual surfaces, were included. Each subject was required to have a whole-mouth mean gingivitis score of ≥1.95, according to the Modified Gingival Index (MGI) [18], and a whole-mouth mean plaque score of ≥1.95, according to the Turesky modification of the Quigley-Hein Plaque index (PI) [19] on six surfaces per tooth [20]. Other study inclusion criteria included the absence of moderate/advanced periodontitis (ADA types III and IV), absence of significant oral soft tissue pathology other than plaque-induced gingivitis; and no fixed or removable orthodontic appliance or removable dentures. Periodontitis patients were excluded based on Tanner’s early periodontitis definition [21], which required one or more periodontal sites with >2 mm periodontal attachment loss in a given subject with mean clinical attachment level ≤ 1.5 mm. Key exclusion criteria included regular use of products such as triclosan dentifrices and mouthrinses containing EO, CPC, or CHX within two weeks prior to baseline; use of antibiotic, anti-inflammatory, or anticoagulant therapy during the study or within two weeks prior to baseline; history of significant adverse effects, including allergies following use of oral hygiene products; known sensitivity to the investigational product ingredients; current, or history of alcohol or drug abuse; and participation in a clinical trial within 30 days of the start of the study.

### Interventions

At baseline, after abstaining from oral hygiene for at least eight hours, but no more than 18 h, pre-screened subjects underwent an oral examination, and gingivitis and plaque assessments. A disclosing dye (0.5% basic fuchsin neutral red solution [Byoformula, Taubate, São Paulo, Brazil]) was used to assess plaque. Eligible subjects received a dental prophylaxis to remove plaque, calculus and stain, and were randomly assigned to one of the three treatment groups:

1) a negative control group, or a Mechanical Oral Hygiene (MOH) only group, received a fluoridated toothpaste (Colgate Cavity Protection, Colgate-Palmolive Company, New York, USA) and REACH® Soft-bristled toothbrush (Johnson & Johnson, New Jersey USA);

2) a test group received the same toothpaste and toothbrush, plus LISTERINE® ZERO™, an Alcohol Free Mouthrinse (AFM) (Johnson & Johnson, New Jersey, USA);

3) a positive control group received the same toothpaste and toothbrush, plus LISTERINE® COOL MINT®, an Alcohol Containing Mouthrinse (ACM) (Johnson & Johnson, New Jersey, USA).

Both AFM and ACM contain a fixed combination of four essential oils [eucalyptol (0.092%), menthol (0.042%), methyl salicylate (0.060%), and thymol (0.064%)], as well as sorbitol, poloxamer, buffer, flavour and dye. AFM also contains Sodium Lauryl Sulphate and propylene glycol.

At the start of the study, all subjects received REACH® Soft-bristled toothbrush, Colgate® anticavity fluoride toothpaste and were instructed to brush with one ribbon of toothpaste in their usual manner twice daily. Colgate anticavity fluoride toothpaste contained Sodium Mono-fluorophosphate (0.76% or 0.15% *w*/*v* Fluoride Ion), Dicalcium Phosphate Dihydrate, Water, Glycerin, Sodium Lauryl Sulfate, Cellulose Gum, Flavor, Tetrasodium Pyrophosphate, Sodium Saccharin. Subjects in the ACM or AFM groups also received the assigned mouthrinse and plastic dosage cups marked at 20 mL level and were instructed to rinse with 20 mL of full strength mouthrinse for 30 s after brushing twice daily. All subjects received diaries to document compliance with the homecare regimen. Diaries were checked monthly and bottles were weighed monthly to match the number of rinses reported in the diaries to the actual used volume. Absence of >3 consecutive rinses or >5 rinses within each 30-day interval between study visits constituted a protocol violation and the subject could be removed from the study at the discretion of the clinical investigator or the Sponsor.

Randomization was based on a block randomization scheme devised by the Biometrics and Clinical Data Systems Department at Johnson & Johnson, with a block size of six. Each subject was assigned a unique randomization number that determined the treatment assigned to that subject according to a randomization schedule. The randomization number was assigned sequentially in ascending order and could not be reassigned to another subject. Investigational supplies were kept separate from the site personnel involved in examining the subjects. The dental examiner was, therefore, blinded to treatment throughout the study period. Subjects received investigational products in blinded packaging, and personnel dispensing the investigational supplies or supervising their use did not participate in the examination of subjects to minimize potential bias. The first rinsing and the rinsing on the examination visits were under supervision at the study site. All other rinsing was unsupervised.

### Assessments and outcomes

Efficacy assessments were performed at baseline and at the one-, three- and six-month visits. This involved visual assessment of gingival inflammation, supragingival plaque, and gingival bleeding, as measured by MGI, PI, and gingival bleeding index (BI) [22, 23], respectively. Subjects were asked to abstain from oral hygiene for at least eight hours, but no more than 18 h prior to their clinical examinations.

A trained and calibrated dental examiner performed all examinations in the following order: oral tissues assessment, MGI, BI and PI. One examiner was used throughout the study.

Gingivitis was assessed using the MGI on the buccal and lingual marginal gingiva and interdental papillae of all scorable teeth as follows: 0 - normal (absence of inflammation); 1 - mild inflammation of any portion of the gingival unit; 2 - mild inflammation of the entire gingival unit; 3 - moderate inflammation of the gingival unit; 4 -severe inflammation of the gingival unit.

To assess gingival bleeding, a periodontal probe was inserted into the gingival crevice, and swept from distal to mesial around the tooth at an angle of approximately 60°, while in contact with the sulcular epithelium. Each of four gingival areas (distobuccal, mid-buccal, mid-lingual, and mesiolingual) around each tooth was assessed. After approximately 30 s, bleeding at each gingival unit was recorded according to the following scale: 0 - absence of bleeding after 30 s; 1 – bleeding after 30 s; 2 – immediate bleeding.

The plaque area was scored on six surfaces per tooth [20] (distobuccal, midbuccal and mesiobuccal, distolingual, midlingual and mesiolingual) of all scorable teeth as follows: 0 - no plaque; 1 - separate flecks or discontinuous band of plaque at the gingival margin; 2 - up to 1 mm continuous band of plaque at the gingival margin; 3 - band of plaque wider than 1 mm but less than 1/3 of surface; 4 - plaque covering 1/3 or more, but less than 2/3 of surface; 5 - plaque covering 2/3 or more of surface.

Safety assessments included oral examinations conducted at baseline, and at monthly visits, to monitor the effect of the mouthrinse formulations on soft and hard tissues. Changes from the baseline and previous visits were recorded at each subsequent clinic visit. Clinically significant findings were recorded as adverse events (AEs) and an assessment was made regarding relationship to investigational product at the discretion of a medically qualified clinical examiner.

During the study, subjects were instructed to follow their usual dietary habits and normal oral care regimen, incorporating only the toothpaste, toothbrush, and mouthrinse provided to them. Subjects were also allowed to continue use of an interdental cleaning device to remove impacted food between the teeth if it was part of their usual oral care regimen. No other oral hygiene procedures were permitted, including teeth cleaning or dental work, except in an emergency.

The primary efficacy endpoint was the mean MGI at six months, while the secondary endpoints included mean MGI at one and three months; and mean PI and BI at one, three, and six months. Safety was assessed by summarizing all AEs considered related to treatment.

### Statistical analyses

The planned sample size of 330 subjects (110 per treatment group) provided 90% power to detect a between-treatment difference of 0.08, assuming a standard deviation [SD] of 0.180, based on previous studies [16, 24] for whole-mouth mean MGI at a 0.05 significance level (two-sided)**.** Demographic and baseline characteristics were compared across treatment groups using analysis of variance (ANOVA), Chi-square test, or Fisher’s exact test. Efficacy analysis was based on the full analysis set (FAS), following the Intent-to-Treat principle, defined as all randomized subjects who used the study product and had baseline and at least one post-baseline data point for mean MGI. Missing data was assumed Missing At Random. No imputation of missing data was performed. Statistical comparisons for primary and secondary variables were based on one-way analysis of covariance (ANCOVA), with treatment as a factor and the corresponding baseline value as a covariate. Comparisons were made in the following sequential manner to control the family-wise error rate of 0.05: ACM versus MOH with respect to mean MGI; AFM versus MOH with respect to mean MGI and; ACM versus AFM with respect to mean MGI. All comparisons were made using a 0.05 level test (two-sided) and secondary endpoints were analysed in the same manner. Safety analysis was based on all randomized subjects who used the study product.

### *Post-hoc* analysis

An exploratory *post-hoc* analysis was conducted to evaluate the non-inferiority (defined as “at least as good as”) of AFM compared with ACM. Using Fieller’s theorem [25, 26], 90% confidence intervals (CIs) for the ratio of observed mean MGI scores of AFM and ACM at months 1, 3, and 6 were constructed. According to ADA guidelines (American Dental Association Council on Scientific Affairs 2011), the criterion for non-inferiority was satisfied when the entire 90% Fieller CI consisted of values no greater than 110%.

## Results

**Fig. 1:**
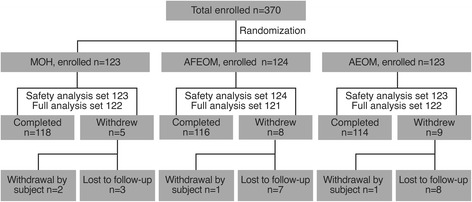
Subject disposition/CONSORT flow diagram. MOH: mechanical oral hygiene; AFM: alcohol-free essential oil mouthrinse; ACM: alcohol-containing essential oil mouthrinse

Of the 370 subjects enrolled in the study and randomized, 348 completed the study, while 22 withdrew or were lost to follow up (Fig. 1). There were no protocol violations leading to removal from the study. In total, 123, 124, and 123 subjects were included in the MOH, AFM, and ACM groups, respectively; 122, 121, and 122 subjects who had both baseline and post-baseline data were included in the FAS. The mean (SD) age of subjects was 36.4 (13.46) years and the majority were females (60.3%), Caucasian (91.1%), and non-smokers (90.5%). Overall, mean (SD) MGI, PI, and BI were 2.452 (0.147), 2.994 (0.241), and 0.618 (0.161), respectively. Baseline and demographic characteristics were comparable between the three study groups (Table 1).

**Table 1:** Subject demographic and baseline characteristics

Parameters	MOH	AFM	ACM	Total	Overall
	(*n* = 123)	(*n* = 124)	(*n* = 123)	(*n* = 370)	*p*-value
Age (years)	38.8 ± 11.75	35.1 ± 14.43	35.2 ± 13.83	36.4 ± 13.46	0.053^a^
Sex n (%)					0.757^b^
Male	50 (40.7)	46 (37.1)	51 (41.5)	147 (39.7)	
Female	73 (59.3)	78 (62.9)	72 (58.5)	223 (60.3)	
Race n (%)					>0.999^c^
White	112 (91.1)	113 (91.1)	112 (91.1)	337 (91.1)	
Black or African- American	1 (<1.0)	1 (<1.0)	1 (<1.0)	3 (<1.0)	
Asian	1 (<1.0)	0	0	1 (<1.0)	
Other	9 (7.3)	10 (8.1)	10 (8.1)	29 (7.8)	
Smoker n (%)					0.876^b^
Yes	13 (10.6)	11 (8.9)	11 (8.9)	35 (9.5)	
No	110 (89.4)	113 (91.1)	112 (91.1)	335 (90.5)	
Whole-mouth scores					
Baseline Mean MGI	2.439 ± 0.147	2.454 ± 0.149	2.462 ± 0.146	2.452 ± 0.147	0.450^a^
Baseline Mean PI	2.976 ± 0.224	2.991 ± 0.228	3.014 ± 0.269	2.994 ± 0.241	0.456^a^
Baseline Mean BI	0.604 ± 0.132	0.628 ± 0.183	0.623 ± 0.163	0.618 ± 0.161	0.474^a^

Data are mean ± SD unless otherwise specified

*ACM* Alcohol-containing essential oil mouthrinse, *AFM* Alcohol-free essential oil mouthrinse, *BI* Bleeding index, *MGI* Modified gingival index, *MOH* Mechanical oral hygiene, *PI* Plaque index, *SD* Standard deviation

^a^*p*-values are based on ANOVA model with term for treatment

^b^*p*-values are based on Chi-square test

^c^based on Fisher’s exact test

### Efficacy

#### Primary endpoint

At six months, ACM use resulted in a 28.2% reduction (between treatment difference [95% CI]; *p* value) (−0.62 [−0.670, −0.570]; *p* < 0.001) in gingivitis, as measured by MGI, compared to MOH. AFM showed a 26.7% reduction (−0.59 [−0.637, −0.538]; *p* < 0.001) in mean MGI versus MOH. However, no significant difference in gingivitis reduction was found between ACM and AFM treatments after six months of use (−0.033 [−0.083, 0.017]; *p* = 0.198) (Table 2).

**Table 2:** Whole-mouth mean modified gingival index (MGI) at months 1, 3, and 6 (full analysis set)

Mean MGI	MOH(*n* = 122)	AFM (*n* = 121)	ACM (*n* = 122)
Baseline			
Mean ± SE	2.438 ± 0.013	2.452 ± 0.013	2.463 ± 0.013
Month 1			
Adj. mean ± SE	2.191 ± 0.014	1.832 ± 0.014	1.838 ± 0.014
Difference between treatments (95% CI)		Percent difference (%)	*p*- value^a^
AFM vs MOH	−0.359 (−0.399, −0.319)	−16.4	<0.001
ACM vs MOH	−0.353 (−0.393, −0.313)	−16.1	<0.001
ACM vs AFM	0.006 (−0.034, 0.046)	0.3	0.756
Month 3^b^			
Adj. mean ± SE	2.257 ± 0.018	1.772 ± 0.018	1.768 ± 0.018
Difference between treatments (95% CI)		Percent difference (%)	*p*- value
AFM vs MOH	−0.486 (−0.535, −0.437)	−21.5	<0.001
ACM vs MOH	−0.489 (−0.539, −0.440)	−21.7	<0.001
ACM vs AFM	−0.004 (−0.053, 0.045)	−0.2	0.878
Month 6^c^			
Adj. mean ± SE	2.201 ± 0.018	1.614 ± 0.018	1.581 ± 0.018
Difference between treatments (95% CI)		Percent difference (%)	*p*- value
AFM vs MOH	−0.587 (−0.637, −0.538)	−26.7	<0.001
ACM vs MOH	−0.620 (−0.670, −0.570)	−28.2	<0.001
ACM vs AFM	−0.033 (−0.083, 0.017)	−2.0	0.198

*ACM* Alcohol-containing essential oil mouthrinse, *AFM* Alcohol-free essential oil mouthrinse, *CI* Confidence interval, *MGI* Modified gingival index, *MO* Mechanical oral hygiene, *SE* Standard error

^a^*p*-values are based on ANCOVA model with term for treatment and baseline value as covariate

^b^At month 3, data for MOH, AFM, and ACM groups were obtained from 119 subjects in each group

^c^At month 6, data for MOH, AFM, and ACM groups were obtained from 118, 116, and 114 subjects, respectively

#### Secondary endpoints

Statistically significant reductions in gingivitis compared to MOH were also seen at one and three months in both the ACM and AFM mouthrinse groups (Table 2). Similar to the results at six months, no significant differences were observed in gingivitis reduction between the two mouthrinse groups at one month (0.006 [−0.034, 0.046]; *p* = 0.756) or three months (−0.004 [−0.053, 0.045] *p* = 0.878) of use.

**Fig. 2:**
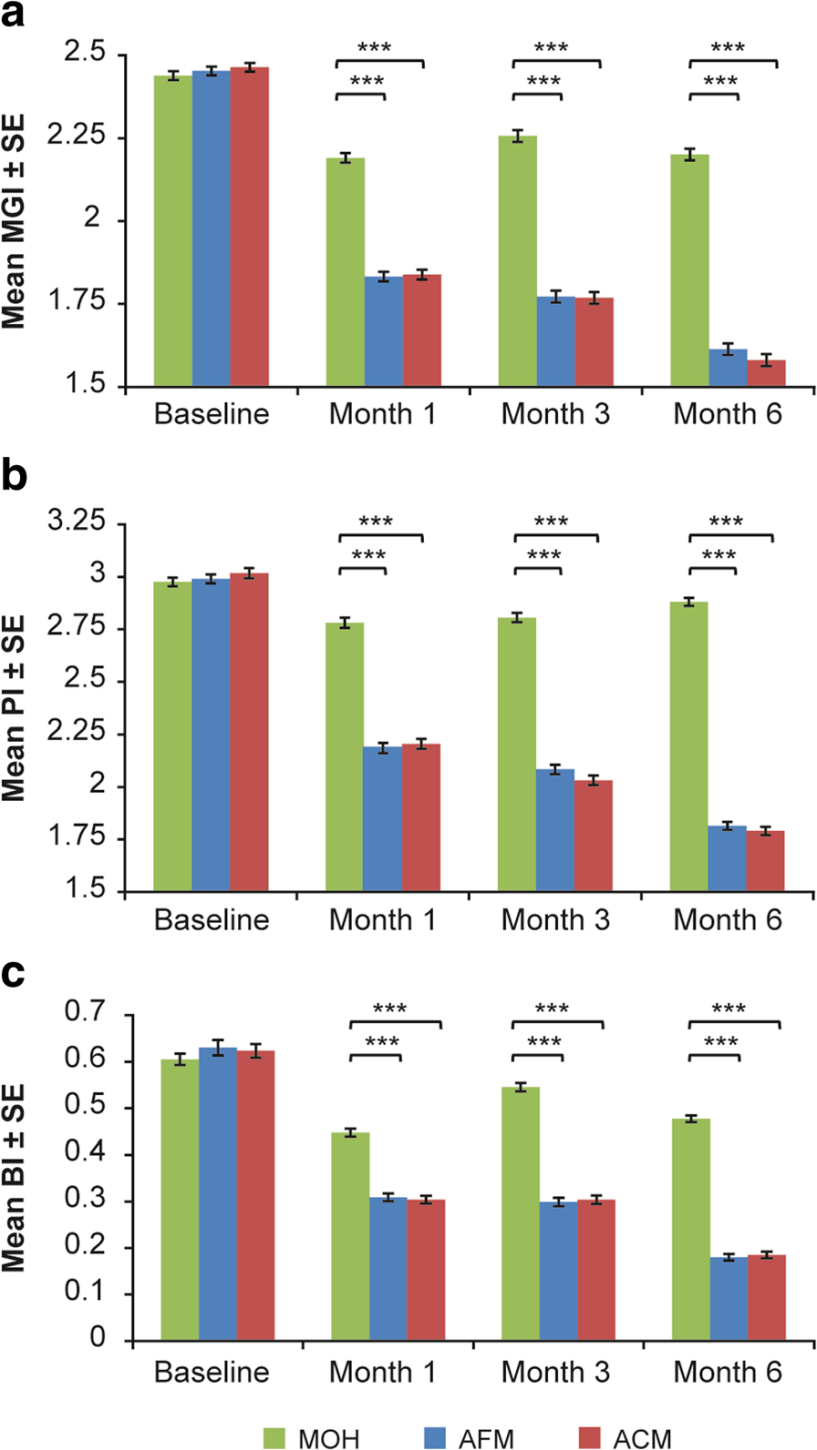
**a** Mean modified gingival index (MGI), **b** plaque index (PI), and **c** gingival bleeding index (BI) at baseline, 1, 3, and 6 months across all treatment groups. Baseline values were based on raw scores, and post-baseline means are based on adjusted means. MOH: mechanical oral hygiene; AFM: alcohol-free essential oil mouthrinse; ACM: alcohol-containing essential oil mouthrinse; SE: standard error; *** *p* < 0.001

Both mouthrinses significantly reduced plaque and gingival bleeding compared to MOH at months one, three, and six (*p* < 0.001) (Tables 3 and 4). No significant difference was seen in the reduction in plaque or gingival bleeding between ACM and AFM groups at one, three, and six months. Figure 2 illustrates the reduction in whole-mouth mean MGI, PI, and BI over the study duration across all treatment groups.

**Table 3:** Whole-mouth mean plaque index (PI) at months 1, 3, and 6 (full analysis set)

Mean PI	MOH (*n* = 122)	AFM (*n* = 121)	ACM (*n* = 122)
Baseline			
Mean ± SE	2.976 ± 0.020	2.991 ± 0.021	3.017 ± 0.024
Month 1			
Adj. mean ± SE	2.781 ± 0.024	2.186 ± 0.024	2.206 ± 0.024
Difference between treatments (95% CI)		Percent difference (%)	*p*- value^a^
AFM vs MOH	−0.595 (−0.662, −0.527)	−21.4	<0.001
ACM vs MOH	−0.574 (−0.641, −0.507)	−20.6	<0.001
ACM vs AFM	0.021 (−0.047, 0.088)	1.0	0.544
Month 3^b^			
Adj. mean ± SE	2.806 ± 0.022	2.084 ± 0.022	2.032 ± 0.022
Difference between treatments (95% CI)		Percent difference (%)	*p*- value^a^
AFM vs MOH	−0.723 (−0.785, −0.661)	−25.8	<0.001
ACM vs MOH	−0.775 (−0.837, −0.712)	−27.6	<0.001
ACM vs AFM	−0.052 (−0.114, 0.010)	−2.5	0.102
Month 6^c^			
Adj. mean ± SE	2.881 ± 0.019	1.815 ± 0.019	1.791 ± 0.019
Difference between treatments (95% CI)		Percent difference (%)	*p*- value^a^
AFM vs MOH	−1.065 (−1.118, −1.012)	−37.0	<0.001
ACM vs MOH	−1.090 (−1.143,-1.036)	−37.8	<0.001
ACM vs AFM	−0.024 (−0.078, 0.029)	−1.3	0.371

*ACM* Alcohol-containing essential oil mouthrinse, *AFM* Alcohol-free essential oil mouthrinse, *CI* Confidence interval, *MGI* Modified gingival index, *MOH* Mechanical oral hygiene, *SE* Standard error

^a^*p*-values are based on ANCOVA model with term for treatment and baseline value as covariate

^b^At month 3, data for MOH, AFM, and ACM groups were obtained from 119 subjects in each group

^c^At month 6, data for MOH, AFM, and ACM groups were obtained from 118, 116, and 114 subjects, respectively

**Table 4:** Whole-mouth mean gingival bleeding index (BI) at months 1, 3, and 6 (full analysis set)

Mean BI	MOH (*n* = 122)	AFM (*n* = 121)	ACM (*n* = 122)
Baseline			
Mean ± SE	0.605 ± 0.012	0.630 ± 0.017	0.623 ± 0.015
Month 1			
Adj. mean ± SE	0.447 ± 0.008	0.309 ± 0.008	0.304 ± 0.008
Difference between treatments (95% CI)		*p*- value^a^	
AFM vs MOH	−0.137 (−0.160, −0.114)	<0.001	
ACM vs MOH	−0.142 (−0.165, −0.119)	<0.001	
ACM vs AFM	−0.005 (−0.028, 0.018)	0.657	
Month 3^b^			
Adj. mean ± SE	0.545 ± 0.009	0.299 ± 0.009	0.304 ± 0.009
Difference between treatments (95% CI)		*p*- value^a^	
AFM vs MOH	−0.246 (−0.271, −0.221)	<0.001	
ACM vs MOH	−0.241 (−0.266, −0.216)	<0.001	
ACM vs AFM	0.006 (−0.019, 0.030)	0.661	
Month 6^c^			
Adj. mean ± SE	0.477 ± 0.007	0.180 ± 0.007	0.185 ± 0.007
Difference between treatments (95% CI)		*p*- value^a^	
AFM vs MOH	−0.297 (−0.316, −0.278)		
ACM vs MOH	−0.292 (−0.311, −0.272)		
ACM vs AFM	0.005 (−0.014, 0.025)		

*ACM* Alcohol-containing essential oil mouthrinse, *AFM* Alcohol-free essential oil mouthrinse, *CI* Confidence interval, *MGI* Modified gingival index, *MOH* Mechanical oral hygiene, *SE* Standard error

^a^*p*-values are based on ANCOVA model with term for treatment and baseline value as covariate

^b^At month 3, data for MOH, AFM, and ACM groups were obtained from 119 subjects in each group

^c^At month 6, data for MOH, AFM, and ACM groups were obtained from 118, 116, and 114 subjects, respectively

### Post hoc analysis

A *post-hoc* statistical analysis was conducted to demonstrate that AFM is “at least as good as” ACM. The ratio of least square means of whole-mouth MGI between AFM and ACM at six months was 1.021 or 102% (90% Fieller CI [99%, 105%]) (Table 5).

**Table 5:** *Post-hoc* analysis: Fieller confidence interval (CI) for whole-mouth mean gingival index^a^

Treatment	Mean MGI		
	Month 1	Month 3	Month 6
AFM	1.832 ± 0.0144	1.772 ± 0.0176	1.614 ± 0.0179
ACM	1.838 ± 0.0144	1.768 ± 0.0177	1.581 ± 0.0181
AFM/ACM (90% CI)	0.997 (0.978, 1.015)	1.002 (0.979, 1.026)	1.021 (0.994, 1.048)

Data are adj. Mean ± standard error. *ACM* Alcohol-containing essential oil mouthrinse, *AFM* Alcohol-free essential oil mouthrinse, *CI* Confidence interval;

^a^Results are based on ANCOVA model with term for treatment and baseline value as covariate

### Safety

All treatments were well tolerated by the subjects. The reported AEs were mild or moderate in severity and no serious AEs were reported, nor did any subject withdraw from the study due to AEs. Three subjects (0.8%) experienced AEs that were judged as related to the study product. This included one subject each in the ACM and AFM groups who experienced sensitivity of teeth, and one subject in the ACM group who reported dyspepsia. All investigation product–related AEs resolved without any treatment.

## Discussion

Historically, a key concern with formulating EO mouthrinses without alcohol was the subsequent reduction in the solubility of these essential oils, leading to their diminished bioactivity and ultimately antimicrobial efficacy. The formulation of an alcohol-free EO mouthrinse therefore required the replacement of alcohol with equally effective solubilisers, while maintaining a balance between all active and inactive ingredients for optimal antimicrobial efficacy.

Contrary to common perception, rinsing with a vehicle that contains alcohol at levels found in mouthrinses, does not provide any clinical benefit. For example, Lamster, et al. [27] demonstrated that a vehicle rinse containing the same amount of alcohol (26.9%) as a marketed ACM performed similarly to rinsing with a water control after six months of use. In that supervised clinical trial, the ACM group had significantly greater reductions in plaque and gingivitis levels when compared to the alcohol vehicle and water control groups. These findings have since been further validated in a systematic review and meta-analysis [28].

To develop the AFM, systematic evaluation of multiple non-alcoholic delivery systems in saliva-derived mixed-species in vitro biofilm models [29] helped identify the precise combination of a sodium lauryl sulphate, poloxamer, propylene glycol, and sorbitol delivery system that was effective in maintaining bioavailability and bioactivity of EOs. At a range of 0.20%–0.35% *w*/w sodium lauryl sulphate, and a total oil phase content of approximately 0.36% w/w, the propylene glycol concentration was found to be optimum between 5% and 13% w/w, while sorbitol was optimum between 10% and 25% w/w (when added as a 70% sorbitol solution) [30, 31] Both AFM and ACM contain a fixed combination of four essential oils [eucalyptol (0.092%), menthol (0.042%), methyl salicylate (0.060%), and thymol (0.064%)], as well as other formulation ingredients.

The first proof-of-principle, human study on AFMs used a two-week, no-oral-hygiene, experimental gingivitis model in which the AFM group showed a reduction in plaque by 23.9% and gingivitis by 10.4%, compared to the negative control rinse group [15]. However, while the results provided an indication of product performance, the restrictions on mechanical oral hygiene limit the applicability of the results.

It is typical to demonstrate the efficacy of a mouthrinse in long-term clinical trials of at least six months in duration, a time period consistent with common recommendations to patients for regular dental visits. A recent meta-analysis identified 35 clinical trials of six-month or longer on EO mouthrinses [7] and included 29 trials that met the inclusion criteria. Two of these 29 studies included an AFM but it was not compared with ACM. One of these two studies was published [16]. In this published six-month study, the efficacy of AFM was assessed in comparison to a negative control mouthrinse and an alcohol-free 0.05% CPC mouthrinse. Subjects performed their daily mechanical oral hygiene (MOH). The EO mouthrinse significantly reduced plaque (31.6%) and gingivitis (24.0%) at six months, compared to the control rinse, and was found to be superior to the CPC mouthrinse. The results of the current study show that both the AFM and ACM provide a statistically and clinically significant additional benefit in subjects who performed their daily MOH. After one month, use of either the AFM or ACM showed significant reduction in plaque (21.4% and 20.6%, respectively) and gingivitis (16.4% and 16.1%, respectively) compared to MOH alone. By month six, further reduction in plaque was noted; up to 37.0% and 37.8% in the AFM and ACM groups, respectively, and gingivitis reduction up to 26.7% and 28.2%, respectively.

No significant difference was found between the ACM and AFM in plaque and gingivitis reduction (*p* = 0.371 and *p* = 0.198, respectively) or in gingival bleeding (*p* = 0.590), reinforcing the evidence that this alcohol-free delivery system was successful in retaining the EO activity to prevent and reduce gingivitis and plaque.

While the *post-hoc* analysis should be judged with the caveat that it was exploratory in nature, the results (90% Fieller CI [99%, 105%]) suggest that since the upper 90% Fieller CI is below 110% as specified by the ADA Guidelines for Determination of Efficacy in Product Evaluation (American Dental Association Council on Scientific Affairs 2011), AFM is at least as good as ACM, adding to the evidence supporting the use of AFM. Thus, the inclusion of either ACM or AFM to an individual’s oral hygiene regimen can play a valuable role in reducing plaque, gingivitis, and gingival bleeding beyond using MOH alone. Overall, improving patients’ oral hygiene may also help to improve gingival inflammation.

A limitation of this study is the absence of a placebo rinse in the MOH control group. This was due to the inability to provide a placebo rinse within the timeframe the clinical site had available to conduct the study. The bias introduced by the placebo effect in clinical trials has been well documented [32, 33], so it is possible that the act of rinsing/not rinsing itself may have had some modest effect on outcomes. However, the results of the MOH group in the current study are similar to results from control groups using mechanical oral hygiene plus placebo mouthrinses in previously published studies [16, 34–37] and other studies have shown that placebo rinses with alcohol were not statistically different from water control rinses.

## Conclusions

Alcohol-free and alcohol-containing EO mouthrinses were able to reduce plaque, gingivitis, and gingival bleeding in comparison to the use of mechanical oral hygiene alone in a six-month, randomized study. No significant differences in efficacy in reducing plaque, gingivitis and gingival bleeding were found between alcohol containing and alcohol free essential oil mouthrinse formulations.

## Abbreviations

ACM: Alcohol-containing mouthrinse
ADA: American Dental Association
AE: Adverse Event
AFM: Alcohol-free mouthrinse
ANCOVA: Analysis of covariance
ANOVA: Analysis of Variance
BI: Bleeding index
CHX: Chlorhexidine
CI: Confidence Interval
CPC: Cetylpyridinium Chloride
EO: Essential Oils
FAS: Full Analysis Set
MGI: Modified Gingival Index
MOH: Mechanical oral hygiene
PI: Plaque index
SD: Standard Deviation
